# A comparative analysis of determinants of low birth weight and stunting among under five children of adolescent and non-adolescent mothers using 2015/16 Tanzania Demographic and Health Survey (TDHS)

**DOI:** 10.1186/s40795-021-00468-6

**Published:** 2021-11-04

**Authors:** Ramadhani H. Mtongwa, Charles Festo, Ester Elisaria

**Affiliations:** 1 The Nelson Mandela Institution of Science and Technology in Collaboration with Ifakara Health Institute, P.O. Box 447, Arusha, Tanzania; 2 Department of Impact Evaluation, Health System and Policy Analysis, Ifakara Health Institute, Plot 463, Kiko Avenue Mikocheni, P.O. Box 78 373, Dar es Salaam, Tanzania

**Keywords:** Low birth weight, Stunting, Adolescents, Under-five, Tanzania

## Abstract

**Background:**

Tanzania is one of the Sub-Saharan African country with nearly 12 out of 60 million people being adolescent. The prevalence of child marriage is higher with one out of every three girls being married before reaching their 18th birthday, 5 % being married by the age of 15, and 31% by the age of 18 years. Literature shows early pregnancy is associated with Low Birth Weight (LBW) and stunting among children under 5 years. This paper explores variation and factors associated with low birth weight and stunting among children born by adolescent and non-adolescent mothers.

**Methods:**

Data from 13,266 women with children under 5 years collected as part of the 2015/2016 TDHS was re-analyzed using STATA version 14 software while accounting for survey design. A total of 6385 women (of which 7.2% were adolescent) and 8852 women (of which 6.7% were adolescent) were involved in the analysis of child birth weight and stunting respectively. Descriptive statistics stratified by maternal age was conducted with LBW and stunting as outcome variables followed by logistic regressions models controlling for confounding variables.

**Results:**

The proportion of obese or overweight adolescent and non-adolescent mothers was 11.8 and 36.5% respectively. Antenatal care (ANC) attendance, areas of residence and social economic status were very similar in the two maternal age groups. Non- adolescent mothers had reduced odds of giving birth to LBW babies compared to adolescent mothers (Adjusted Odds Ratio (AOR) = 0.34; 95% CI: 0.22–0.50). Maternal undernutrition (AOR = 2.29; 95% CI: 1.43–3.67), being divorced, separated or widowed (AOR = 1.76; 95% CI: 1.24–2.50) and having at least four ANC visits (AOR = 0.64; 95% CI: 0.49–0.83) were significantly associated with reduced odds of having a LBW. Child stunting was not associated with maternal age. Maternal high socioeconomic status (AOR = 0.69; 95% CI: 0.57–0.84) and maternal obesity or overweight (AOR = 0.77; 95% CI: 0.64–0.92) were negatively associated with stunting. Child birth weight, sex, and age were significantly associated with stunting.

**Conclusion:**

Maternal age was a predictor of LBW but not stunting. ANC attendance and not living with a spouse increase the risk of LBW babies. Stunting was associated with low maternal body mass index (BMI), low socioeconomic status, child birth weight, gender, and age. A multi-sectoral approach is needed to address child nutrition problems with teenagers ‘specific intervention that offer emotional support, and health education during pregnancies for improving immediate and later life child birth outcomes.

## Background

Globally about 1.2 billion people are adolescents aged 10–19 years [1] with Sub-Saharan Africa contributing to 23% of the global population of the adolescents [2]. From 2010 to 2030 the expected top five countries in Sub-Saharan Africa with the greatest number of adolescents are Nigeria (9.2 million), the United Republic of Tanzania (3.7 million), the Democratic Republic of the Congo (3.3 million), Uganda (2.5 million) and Kenya (2.3 million) [3].

Tanzania has a population of approximately 60 million people of which 12 million are adolescents aged 10–19 years [4]. The country has made a significant progress in improving child nutrition status during the period 1992–2015 whereas stunting declined from 50 to 34% [5]. This still higher compared to that of global level 24.4% [23.9–24.8] [6] and lower compared to that of the Africa region (32%) [7]. Despite these gains there have been little changes in adolescent health indicators. Almost one out of every three girls is married before her 18th birthday [8]. Five percent of girls are married by the age of 15 years, and 31 % are married by the age of 18 years [8]. Despite early marriage, one out of three adolescents are anaemic and one out of four are stunted [8]. Maternal malnutrition is a primary factor of foetal growth retardation in poorer population groups, and pregnant adolescents are at even higher risk [9]. Some literatures also revealed factors like lower level of maternal education, poor access to health service, poor complementary feeding practices, poor living conditions, maternal and child age being associated with childhood stunting [10–12].

Childbearing during adolescence is linked to a number of challenges. Teen mothers often have less structural resources, less educated, and lack financial independence as compared to adult mothers in both high, low, and middle-income countries [12]. Often, young mothers are likely to have low maternal weight gain during pregnancy and anemia due to competing needs of nutrients during pregnancy [13]. This explains why adolescent mothers are likely to give birth to preterm, low-birth-weight babies and with an increased likelihood of infant deaths compared to those born with mothers aged 20 years and above [14]. Furthermore, the high rates of teenage pregnancy contribute significantly to the high prevalence of malnutrition among adolescents themselves and their under-five children [15]. Adolescent mothers are not only challenged by the physical threats to their health, but also socially [16]. Majority of them have not completed their education and consequently have a limited capacity to secure jobs and sustain their livelihood and their children too [14]. They also lack important knowledge on child care which may contributes to childhood malnutrition and hence creating a vicious circle of malnutrition and poverty [17].

Overall, it is estimated that 15 to 20% of all births worldwide are LBW, representing more than 20 million births a year [18]. The estimated prevalence of LBW in Sub Saharan Africa is 13% although 54% of all the children are not weighed at birth [18]. In Tanzania the prevalence of LBW babies is 10%. Maternal age, Body Mass Index (BMI), ANC attendances and place of residence are among factors reported by the literatures as risk factors for LBW babies [12–14].

Despite the overwhelming evidence on adolescent social and economic vulnerabilities, little is known about how maternal age contributes to low birth weight and childhood stunting in Tanzania. This paper explored variation and factors associated with LBW and stunting among children born by adolescent and non-adolescent mothers using data collected as part of the 2015/2016 Tanzania Demographic and Health Survey (TDHS) [5].

## Methods

Secondary data analysis of the 2015/2016 TDHS was done focusing on adolescent and non-adolescent mothers aged 15–49 years with their children 0–59 months. The TDHS is a national representative cross-sectional survey done after every 5 years to monitor and evaluate population health, nutrition, and maternal indicators including feeding practices and health care seeking behaviors. It contained information collected from 13,400 households located in Zanzibar and mainland Tanzania. The survey employed a two-stage stratified sampling design with the first stage involving independent sampling of 59 strata by population proportion to size (PPS). The second stage involved random sampling of households located in each of the selected cluster. The detailed sampling procedures has been published in the 2015/2016 TDHS report [5].

All women aged 15–49 who were usual residents or slept in the selected households the night before the survey were eligible for the interview. Anthropometric measurements (height and weight) of child and that of caretakers were taken at household level on the day of survey. Weight measurements were obtained with an electronic SECA 874 flat scale designed for mobile use and Height was measured with a Shorr board measuring board. Children younger than 24 months or shorter than 85 cm were measured lying down on the board (recumbent length) and standing height taken for older or taller children.

### Data management and analysis

The 2015/2016 TDHS dataset was downloaded from the DHS website after completing the registration and fulfillment of the conditions related to the protection of sensitive data. A total of 13,634 eligible women were identified from 1782 households sampled. Of interviewed women, 27% (2904) were adolescents aged 15–19 years. Analysis was conducted in a sample of women who had a live birth 5 years preceding the survey using STATA version 14 software accounting for survey design. A total of 8852 women (600 adolescent and 8252 non adolescent) with child anthropometric data and 6386 women (459 adolescent and 5927 non adolescent) with child birth weight data was analyzed.

Variable were categorized as per DHS guideline with the normative category used as a reference group in regression analysis [19]. Maternal nutritional status was assessed using the BMI classified as undernutrition (bmi < 18.5), normal (> 18.5 bmi < 25), overweight (> 25 bmi < 30) and obese (bmi > 30). Stunting and child birth weight of under-five children born by adolescents (15–19 years) and non-adolescent mothers (19–49 years) were considered as primary and secondary outcome respectively [20]. Stunting as an outcome variable was prepared using the 2006 World Health Organization (WHO) growth standards reference points based on z-scores <− 2 standard deviation [21]. A child weighted below 2500 g at birth was termed as low birth weight baby. Maternal age was categorized as adolescent (15–19 years) and non-adolescent (20–29, 30–39 and 40–49 years). The household wealth index was developed based on the household’s assets ownership using the principal component analysis. The five equal categories used in the TDHS reports were further categorized into two groups, with the first three being in the low economic status to reflect the actual Tanzania condition.

Frequencies and percentage were used to presents results from descriptive analysis stratified by outcome variables. Univariate logistic regression models were fitted independently with stunting and birth weight as outcome variables while adjusting for socioeconomic and demographic characteristics such as age, height, weight (for both mother and children), maternal characteristics (education, place of residence) and uptake of maternal care. As anticipated, logistic regression analysis was not done separately for each maternal age (adolescent and adult mothers) due to small sample size particularly in the adolescent population. A pooled analysis was done instead for each outcome variable with maternal age as a main exposure. A forward selection procedure was applied during modelling with variable selection based on change in exposure effect estimate. The procedure involved four main steps: a) descriptive analysis and preliminary investigations for association between variables while paying attention to the sizes of effects as well as *p*-values at 95% significant level. b) Variable selection; from prior knowledge, child sex was considered as forced variable in the model and maternal employment was ignored as it was highly correlated with social economic status. One variable at a time from a list of candidate variables obtained from a univariate analysis was then included in the model with and without adjustment of forced variables to help understanding the effect of forced variables. The choice of the “best” predictor to be included in the model was then decided based on the change in exposure effect estimate. Each time a new variable was added in the model, evidence of confounding and multicollinearity was assessed by comparing the effect estimates and standard errors between the “univariate” and “multivariate” models estimates c) Multivariable model were fitted by adding explanatory variables that were removed from the models in step “b” one at a time to helps exploring their effect when added to the model in presence of other variables in the model. Variables that resulted into positive changes in the mean square error were then included in the model. The process was repeated until all variables that provided precise estimates of exposure variables were selected. The final model includes maternal age, marital status, maternal BMI, number of ANC visits, and child sex.

### Ethical considerations

Protocol, study procedures and questionnaires for DHS surveys was reviewed and approved by Institutional Review Board (IRB) and by the National Medical Research committee of Tanzania prior to the 2015 data collection. Before the interviews, written informed consent was obtained from all participants. For illiterate population, a written consent form was presented orally to them in the presence of legally authorized representative or witness such as a family member, friend, or someone independent of the research team. A parent or guardian provided consent for the child or adolescent to be involved in the study. Interviews were performed as privately as possible with each eligible respondent interviewed in the absence of another person.

## Results

### Prevalence of child low birth weight (LBW) among adolescent and non-adolescent mothers

Overall, 14.3% of adolescents and 6.3% of non-adolescent mothers gave birth to low-birth-weight babies (Table 1). By education level, 19% of adolescent mothers with no education had a low-birth-weight babies compared to 7.1% of non-adolescent mothers. The proportion of women who gave birth to LBW babies were very similar across age group and economic status but a bit higher among unemployed adolescent mothers. Urban community appears to have higher proportion of mothers who gave birth to LBW babies, 15.5% for adolescent mothers and 6.9% for adult’s mothers when compared to those located in the rural settings. Twenty-two percent of divorced/separated or widowed adolescent mothers gave birth to LBW babies compared 9.5% found among adult women. Twenty-four percent of the undernourished adolescent mothers gave birth to LBW babies as compared to 10.4% of undernourished adult mothers.

**Table 1:** Prevalence of Child Low Birth Weight among adolescent and non-adolescent mothers by maternal characteristics (*N* = 6386)

Variable	Adolescent mothers (15–19 years.)		Non-Adolescent mothers (20+ years.)	
	N	LBW children n (%)	N	LBW children n (%)
**Overall**	459	66 (14.3)	5927	376 (6.3)
**Level of education**				
No education	34	7 (19.1)	831	59 (7.1)
Primary education	354	52 (14.9)	3867	241 (6.2)
Secondary education or higher	71	7 (0.9)	1229	76 (6.2)
**Wealth quintile**				
Low SES	275	40 (14.4)	3016	186 (6.2)
High SES	184	26 (14.1)	2911	190 (6.5)
**Place of residence**				
Urban	148	23 (15.5)	2255	156 (6.9)
Rural	311	43 (13.7)	3672	220 (6.0)
**Marital status**				
Never married	128	19 (14.9)	330	25 (7.6)
Married or living together	287	37 (12.9)	4842	279 (5.8)
Divorced/ Separated/ Widowed	44	10 (22.0)	755	72 (9.5)
**Maternal BMI**^a^				
Undernutrition	43	11 (23.9)	309	33 (10.4)
Normal	363	46 (12.7)	3646	232 (6.4)
Overweight or obese	53	9 (18.0)	1936	110 (5.7)
**ANC visits**				
Ever attended	200	31 (15.7)	1837	124 (6.7)
Attended at least four visits	218	31 (14.3)	2500	106 (4.3)

*SES* Socio-economic status, *BMI* Body Mass Index, *ANC* Antenatal care, *LBW* Low Birth Weight

*N* Total number of children with birth weight data, *n* Number of children with Low Birth Weight

36 Non-adolescent women had missing height or weight measurement

### Prevalence of child stunting among adolescent and non-adolescent mothers

The proportion of stunted children born by adolescent and non-adolescent mothers was 30.6 and 34.2% respectively (Table 2). Thirty-eight percent of under-five children born by women who have not been to school or with primary level of education were stunted. Over 30 % of children born by mothers living in rural community or within the lower social economic group were stunted. The proportion of stunted children by marital status was very similar, ranging from 25.8 to 32.3% among those born by divorced or separated adolescent women and from 32.2% among those never married to 39.4% among divorced or separated non-adolescent women. Thirty four percent of the under-five stunted children were born by adolescent mothers who were overweight or obese as compared to 26.6% of adult mothers. Adult mothers who were ever attended ANC visit had high percentage (33.9%) of stunted children as compared to adolescent mothers (28.7%).

**Table 2:** Prevalence of child stunting among adolescent and non-adolescent mothers by maternal characteristics (*N* = 8852)

Variable	Adolescent mothers (15–19 yrs)		Non-Adolescent mothers (20+ yrs)	
	N	Stunted children n (%)	N	Stunted children n (%)
**Overall**	600	183 (30.6)	8252	2820 (34.2)
**Level of education**				
No education	79	30 (38.0)	1820	709 (38.9)
Primary education	455	139 (30.6)	5258	1839 (35.0)
Secondary education or higher	66	14 (21.5)	1174	272 (23.2)
**Wealth quintile**				
Low SES	419	137 (32.8)	5403	2124 (39.3)
High SES	181	46 (25.4)	2849	695 (24.4)
**Place of residence**				
Urban	141	36 (25.8)	2148	532 (24.8)
Rural	459	147 (32.0)	6104	2287 (37.5)
**Marital status**				
Never married	141	38 (26.9)	333	107 (32.2)
Married or living together	416	134 (32.3)	7041	2367 (33.6)
Divorced/ Separated/ Widowed	43	11 (25.8)	878	345 (39.4)
**Nutrition Status (BMI)**				
Undernutrition	54	16 (30.0)	552	220 (39.9)
Normal	481	145 (30.2)	5528	2024 (36.6)
Overweight or obese	65	22 (34.3)	2155	573 (26.6)
**ANC visits**				
Ever attended	284	81 (28.7)	2914	989 (33.9)
Attended at least four visits	260	76 (29.1)	3001	913 (30.4)

*SES* Socio-economic status, *BMI* Body Mass Index, *ANC* Antenatal care, *N* Total number of children with anthropometric measurement, *n* Number of stunted children

17 Non- adolescent mothers have missing height or weight measurements

### Predictors of low birth weight

This analysis was conducted in a sample of 6385 women that had a live birth five years preceding the survey and weight data for their most recent child aged. Maternal age (adolescent and non-adolescent mothers) was considered as a main exposure as explain in the method section. Overall, non-adolescent mothers have reduced odds of giving birth to LBW babies when compared to adolescent mothers in both univariate and multivariate analysis. The odds of delivering a LBW babies decreases from 0.41 (95% CI: 0.29–0.57) in crude analysis to 0.34 (95% CI: 0.22–0.50) in adjusted analysis (Table 3). Maternal BMI, marital status and attending at least four ANC visit were significantly associated with LBW. The proportion of LBW babies was higher among mothers with primary education (66.5%), married or living with their partner (71.6%) and those with low socioeconomic status (51.1%).

**Table 3:** Factors associated with Low Birth Weight (*N* = 6386)

	N	n (%)	OR (95% CI)	^a^AOR (95%CI)
**Maternal age (Years)**				
Adolescents (15–19)	459	66 (14.3)	1	1
Adults (20 +)	5927	376 (6.3)	0.41 (0.29–0.57)	0.34 (0.22–0.50)
**Level of education**				
No education	866	65 (14.8)	1	
Primary education	4220	294 (66.5)	0.91 (0.63–1.32)	
Secondary or higher education	1300	83 (18.7)	0.83 (0.55–1.26)	
**Wealth quintile**				
Low SES	3292	226 (51.1)	1	
High SES	3094	216 (48.9)	1.02 (0.78–1.33)	
**Marital status**				
Never married	458	44 (9.8)	1.62 (1.05–2.49)	1.23 (0.75–2.04)
Married or living together	5129	316 (71.6)	1	1
Divorced/ Separated/ Widowed	799	81 (18.4)	1.72 (1.23–2.42)	1.76 (1.24–2.50)
**Place of residence**				
Urban	2402	179 (40.4)	1	
Rural	3984	263 (59.6)	0.88 (0.67–1.16)	
**Maternal BMI**				
Undernutrition	352	42 (9.6)	1.84 (1.23–2.75)	2.29 (1.43–3.67)
Normal	4009	279(63.2)	1	1
Overweight or obese	1989	120 (27.2)	0.86 (0.64–1.14)	0.99 (0.71–1.38)
**At least 4 ANC visits**				
No	2037	155 (53.0)	1	1
Yes	2718	138 (47.0)	0.65 (0.50–0.83)	0.64 (0.49–0.83)
**Child Sex**				
Male	3266	197 (44.5)	1	1
Female	3120	245 (55.5)	1.33 (1.07–1.68)	1.31 (0.98–1.75)

*N* Total number of respondents, *n* Number of respondents with low-birth-weight babies, *%* row percent, *AOR* Adjusted odds ratio, *OR* Odds ratio, *CI* 95% Confidence interval, *SES* Socioeconomic status, *BMI* Body mass index, *ANC* Antenatal care

^a^ Adjusted for maternal age, marital status, maternal BMI, ANC attendance and child sex, 36 Non-adolescent women had missing height or weight measurement

### Predictors of stunting in under-five children

A total of 8852 women with a live birth 5 years preceding the survey who had their height and age measurement documented in the dataset were considered in this analysis. As for LBW analysis, maternal age was considered as the main exposure while controlling for maternal factors: age, education, wealth index, marital status, place of residence, occupation and nutrition status and child characteristics: Birth weight, sex and age in the model. Although, 34.2% of children born by non-adolescent mothers were stunted compared to 30.6% of adolescent mothers, the adjusted analysis did not show any significant difference between these two age groups, AOR: 0.97 (95% CI: 0.73–1.29) (Table 4).

**Table 4:** Factors associated with stunting (*N* = 8852)

	N	n (%)	OR (95% CI)	^a^AOR (95% CI)
**Maternal age (years)**				
Adolescent (15–19)	600	183 (30.2)	1	1
Adults (20+)	8252	2820 (34.2)	1.18 (0.96–1.45)	0.97 (0.73–1.29)
**Level of education**				
No education	1900	739 (24.6)	1	1
Primary education	5712	1978 (65.9)	0.83 (0.73–0.94)	1.05 (0.85–1.30)
Secondary education or higher	1240	286 (9.5)	0.47 (0.38–0.58)	0.81 (0.61–1.08)
**Wealth quintile**				
Low SES	5822	2262 (75.3)	1	1
High SES	3030	742 (24.7)	0.51 (0.45–0.58)	0.69 (0.57–0.84)
**Marital status**				
Never married	475	145 (4.8)	0.87 (0.70–1.10)	1.25 (0.95–1.67)
Married or living together	7457	2501 (83.3)	1	1
Divorced/ Separated/ Widowed	920	357 (11.9)	1.25 (1.04–1.49)	1.19 (0.93–1.51)
**Place of residence**				
Urban	2289	569 (18.9)	1	1
Rural	6563	2435 (81.1)	1.78 (1.48–2.15)	1.21 (0.93–1.56)
**Maternal BMI**				
Undernutrition	606	236 (7.9)	1.13 (0.93–1.38)	1.14 (0.85–1.52)
Normal	6009	2169 (72.3)	1	1
Overweight or obese	2220	596 (19.9)	0.65 (0.56–0.75)	0.77 (0.64–0.92)
**Child characteristics**				
**Birth weight**^b^				
Normal	5245	1568 (90.4)	1	1
Low birth weight	341	166 (9.6)	2.23 (1.67–2.98)	2.40 (1.80–3.20)
**Sex**				
Male	4490	1631 (54.3)	1	1
Female	4362	1372 (45.7)	0.80 (0.72–0.89)	0.77 (0.67–0.89)
**Age (months)**				
0–11	1989	321 (10.7)	1	1
12–23	2065	781 (26.0)	3.17 (2.66–3.76)	3.19 (2.49–4.08)
24–59	4798	1901 (63.3)	3.41 (2.94–3.96)	3.36 (2.76–4.11)

*N* Total number of respondents, *n* Number of under-five children stunted, *%* row percent *AOR* Adjusted odds ratio, *OR* Odds ratio, *CI* 95% Confidence interval, *SES* Socioeconomic status, *BMI* Body mass index, *ANC* Antenatal care

^a^adjusted for maternal level of education, wealth index, marital status, place of residence, occupation, BMI, child birth weight, sex and age

^b^ Done only for children with birth weight data (3263 child were excluded)

In a multivariate analysis, socioeconomic status (SES) was significantly associated with childhood stunting (AOR = 0.69; 95% CI: 0.57–0.84) with children found in higher socioeconomic group having higher odds of being stunted compared to those in lower socioeconomic group. Children born with low birth weight were 2.4 times more likely to be stunted (95% CI: 1.80–3.20) compared to those born with normal birth weight. Female children were less likely to be stunted (AOR = 0.77; 95% CI: 0.67–0.89) when compared to male. Also, children whose age were beyond 1 year had 3 times high odds of being stunted as compared to those with less than 1 year. Further, maternal body structure was significantly associated with child stunting. Children born with overweight or obese mothers had reduced odds of being stunted (AOR = 0.77; 95% CI: 0.64–0.92) when compared to mothers with normal BMI (Table 4).

## Discussion

This study explored factors associated with variation in proportional of Lower Birth Weight babies and stunted children among adolescent and non-adolescent mothers. Our findings showed that adolescent mothers have increased odds of giving birth to LBW babies with no difference in the likelihood of having a stunted child between these two maternal age groups. Unplanned pregnancies, biological immaturity, poor nutrition status during pregnancy are among factors that aggravated to LBW among adolescent mothers [22]. We also found significant increase in risk of LBW to mothers who were divorced/separated/widowed compared to those married. This is similar to the finding reported by Tessema et al. [20], on a study conducted in sub-Saharan Africa and contradict with the studies conducted by Talie et al. [23], and Tshotetsi et al. [24], who found no significant association between marital status and LBW. Lack of social and economic support and stress have been reported as factors associated with delivering LBW infants among single and separated/divorced/widowed mothers [25]. This study found significantly high odds of LBW among undernourished mother as compared to normal ones, a finding similar to that of studies conducted in India [26] and Ethiopia [27]. Factors like poor socio-economic status, unemployment, poor dietary intake and poor weight gain during pregnancy could be the ones associated with delivering LBW infants. Optimal nutrition is particularly critical for pregnant teens [28]. According to WHO guideline a minimum of four ANC visits are required for safe maternity [29], our study has found mothers who attended at least four ANC had lower risk of delivering LBW infants compared to those who attended less than four ANC. This is similar to the finding from a study conducted in India by Priyanka D [30].

The findings showed that maternal age was not significantly associated with childhood stunting. Although children born to adult mothers have slightly high odds of being stunted compared to those belong to adolescent mothers. This finding look similar to that of Agedew et al., done in Ethiopia [31] but is contrary to the finding of Chirande et al., done in Tanzania [32] and Habyarimana et al., done in Rwanda [33]. We found negative association between children whose mothers lived in high socioeconomic status and stunting. This is consistency to the study done in India and sub Saharan Africa [31, 34]. Household purchasing power, sanitation and health insurance are among factors that can contribute to poor or good nutrition status among under five children [32]. Malnutrition is a condition that is associated with poverty since it comes with hunger and lack of food at the right quantity and quality [35–37]. Unlike the study conducted by Francois R. et al. [35], we found stunting is less among obese or overweight mother. In our finding, a unit increase in Body Mass Index, stunting among under-five children of obese or overweight mothers decreased by 23%. This is absolutely higher compared to what reported by Amaha N and Woldeamanuel B [36]; a unit increase in Body Mass Index reduced the odds of U-5 stunting by 4%.

We also looked an association between child characteristics (birth weight, sex and age) and stunting. Our findings showed that children who were born with LBW had two times high chance of being stunted compared to those born with normal weight. This is in line with the studies conducted in Rwanda, Indonesia and Ethiopia [38, 39]. LBW is a predisposing factor for growth attainment [39]. Our study found, stunting in female children decreased by 23% as compared to male child. This agrees with studies conducted in Tanzania, Afghanistan and Ghana [40–42] which showed that boys were disadvantaged in term of stunting status than girls. Inadequate amount of food, culture preference and physical activeness might be the leading factors for stunting among male children compared to girls. We also found high odds of stunting as child age increases. This agrees with the study conducted in Tanzania [41] which showed that the risk of stunted increases as children approach their second year of life. Suboptimal breastfeeding (specifically, non-exclusive breastfeeding) and complementary feeding that is limited in quantity, quality and variety could be the factors that increases risk of stunting on children as age increases [42].

## Conclusion

We found LBW was significantly associated with maternal age with adolescent mothers being at higher risk of giving birth to LBW babies as compared to non-adolescent mothers. Maternal ANC attendance and being divorced, separated or widowed were also showed significantly association with LBW. The findings also show stunting is not associated with maternal age even after controlling for socioeconomic status, maternal nutrition status and child characteristics (birth weight, sex and age). A multi-sectoral approach is needed in addressing child nutrition problems with teenagers ‘specific intervention that offer emotional support, and health education during pregnancies for improving immediate and later life child birth outcomes.

### Limitation of the study

Variability in availability of birth weight data might have biased the results since only 64% of live births had birth weight measurement. The study has a sample of 13,266 women of which only 2904 were adolescent mothers. The unequal sample size by maternal age might have affected the statistical power of the study as some of the estimates may be over or under-reported. Since the this was secondary analysis of the data, some important clues might be missing concerning the causal relationship of dependent factors and outcome. For example, the study revealed out that boys were more stunted than girls but the reasons behind this are incomprehensive. The detailed list of strength and weakness of the DHS dataset have been documented by Corsi et al. [43].

### Clinical implications of the study

The finding reflects the needs of a multi-sectoral approach in addressing child nutrition problems with teenagers ‘specific intervention that offer emotional support, and health education before and during pregnancies for better child birth outcomes. Stakeholders and policy makers are likely to use these findings for changing policies and designing of community related intervention that target adolescent and non-adolescent mothers.

## Data Availability

The study datasets are available from the DHS website through (https://dhsprogram.com/data/available-datasets.cfm).

## Abbreviations

ANC: Antenatal Care
BMI: Body Mass Index
DHS: Demographic and Health Survey
LBW: Low Birth Weight
NBS: National Bureau of Statistics
SES: Social Economic Status
TDHS: Tanzania Demographic and Health Survey
TNNS: Tanzania National Nutritional Survey
UNFPA: United Nation Population Fund
UNICEF: United Nation Children’s Fund
URT: United Republic of Tanzania
WHO: World Health Organization
